# Operationalizing and Evaluating Synchronous Virtual Group Health Interventions: Wide-Scale Implementation at a Tertiary Care Academic Hospital

**DOI:** 10.2196/29841

**Published:** 2022-04-07

**Authors:** Neesha Hussain-Shamsy, Ian McMillan, Sheridan Cook, Alyssa Furfaro-Argier, Andrea Sadler, Faith Delos-Reyes, Lori Wasserman, Sacha Bhatia, Danielle Martin, Emily Seto, Simone N Vigod, Juveria Zaheer, Payal Agarwal, Geetha Mukerji

**Affiliations:** 1 Institute of Health Policy, Management and Evaluation University of Toronto Toronto, ON Canada; 2 Centre for Global eHealth Innovation Techna Institute University Health Network Toronto, ON Canada; 3 Institute for Health System Solutions and Virtual Care Women's College Hospital Toronto, ON Canada; 4 Women's Virtual Women's College Hospital Toronto, ON Canada; 5 Gevity Consulting Inc Toronto, ON Canada; 6 Department of Information Management and Technology Women's College Hospital Toronto, ON Canada; 7 Department of Psychiatry Women's College Hospital Toronto, ON Canada; 8 Department of Cardiology Women's College Hospital Toronto, ON Canada; 9 Reproductive Life Stages Program Women's College Hospital Toronto, ON Canada; 10 Institute for Clinical Evaluative Sciences Toronto, ON Canada; 11 Temerty Faculty of Medicine University of Toronto Toronto, ON Canada; 12 Centre for Addiction and Mental Health Toronto, ON Canada; 13 Family Practice Health Centre Women's College Hospital Toronto, ON Canada; 14 Department of Medicine Women's College Hospital Toronto, ON Canada

**Keywords:** virtual care, group therapy, patient education, videoconferencing, sustainability, innovation, health systems, health promotion, patient portal, electronic medical records, health service delivery, video call

## Abstract

Group-based health interventions are an important component of health promotion and management. To provide continuity of care throughout the COVID-19 pandemic, our institution undertook a rapid pivot to delivering group-based health interventions via a videoconferencing service which was securely embedded into both the electronic medical record and the patient portal to sustainably address immediate health service delivery needs during the pandemic and beyond. In this paper, we (1) describe the institutionally driven operationalization of a system to provide integrated synchronous video group visits across our hospital and (2) present a proposed strategy to comprehensively evaluate outcomes regarding their implementation, quality, and impact. Lessons for other institutions and the potential future role of synchronous video group visits to enhance how care can be scaled for delivery are discussed.

## Introduction

### Background

Group-based health interventions, where small groups of patients receive the same health intervention (eg, patient education or therapy) from one or more facilitators, are frequently used for health promotion and the self-management and treatment of many physical and mental illnesses [1-6]. Group-based health interventions are cost-effective as they allow multiple individuals to receive treatment simultaneously [1,4,7], particularly in settings with a limited supply of qualified providers [1,8].

With the conversion of many health care services to virtual care during the COVID-19 pandemic, the need for virtual approaches to group-based health interventions suddenly became pressing to ensure continuity of care. With little evidence to drive this shift [9], it was not uncommon for technologies and procedures that addressed immediate needs within the context of the pandemic, but which would not necessarily be sustainable in the long-term or optimized for high-quality care, to be used [10]. A systematic review (which included 15 studies of mixed quality) on the use of group videoconferencing for health interventions showed that it was satisfactory to patients with high attendance rates, but had mixed results in terms of its impact on health outcomes; high-quality studies in this review demonstrated positive results in terms of group process outcomes (eg, group cohesion and social support) [9]. The main limitations were that the studies in the review were typically pilot studies and the technical equipment used was supplied by the study, limiting the generalizability of the findings to institutionally driven and scalable approaches to deliver care in the long-term [9]. It is generally accepted that, after the pandemic, virtual care will take a more prominent and permanent place in the health care system. Therefore, it is important for health institutions to address the need for sustainable high-quality solutions with the ability to monitor impact.

This paper aims to reflect on and share the experience of Women’s College Hospital (WCH) in developing and operationalizing a sustainable model of synchronous group video visits during the pandemic. We also present a proposed evaluation strategy that will be used to monitor and evaluate its impact, quality, and outcomes. This will aid other organizations when considering how to plan and move toward sustainable group video visit practices as postpandemic health system planning becomes a priority.

## Methods

### Institutional Context

WCH is an ambulatory tertiary care facility in Toronto, Canada, and is fully affiliated with the University of Toronto. Housed within the hospital is the WCH Institute for Health System Solutions and Virtual Care, a “living laboratory” for developing, testing, and implementing virtual solutions to improve health [11]. In December 2019, the hospital launched Women’s Virtual (WV) [12], an institutional strategic initiative to systematically address virtual care barriers and facilitate a coordinated and widespread adoption of virtual care across the organization, with the introduction of individual video visits.

WCH offers several evidence-informed group health interventions, which were historically delivered solely in person. The groups range from 10 to 25 patient participants, span an array of health concerns (eg, mental health, chronic pain management, and cardiac rehabilitation), and offer a variety of health interventions (eg, therapy, education, and exercise). Shortly after the ramp-down of nonemergent in-person health care visits in March 2020 at the pandemic’s onset, 5 divisions across WCH identified the urgent need to ensure continuity of care by delivering these group interventions virtually. The eventual implementation of virtual group health interventions was part of the WV strategic roadmap, but these events increased its priority as an initiative for the hospital to develop in a manner that both addressed the immediate needs triggered by the pandemic and the long-term need for a program of sustainable virtual care.

By April 2020, because of its ability to replicate elements of in-person groups (eg, immediate and reciprocal visual cues, facial expressions, and the ability to support conversational dialogue), hospital leadership, in consultation with relevant departments and providers, determined that synchronous videoconferencing would be the best option to use in pursuit of the continued delivery of group health interventions.

### System Development and Operationalization

In line with the hospital’s strategic mandate, the development and implementation of a system to deliver synchronous video group health interventions to patients would need to also serve the larger interest of building a sustainable virtual program of care. Therefore, the use of either an existing platform (ie, Zoom [Zoom Video Communications Inc], a videoconferencing service that can be licensed for secure use for health care purposes) or an alternative hospital vendor either external to or integrated within the hospital’s existing electronic medical record (EMR; ie, Epic [Epic Systems Corp]) was considered. A process to select a technical platform was conducted to support implementation that would meet security, privacy, and quality considerations beyond a pandemic context. Multiple factors were considered (Textbox 1). Stakeholders decided to move forward with integrated Epic-Zoom group video visits as a long-term sustainable solution that could continue to be used post pandemic. While this approach provided many benefits, there were few previous examples of Epic-Zoom integration for synchronous video group visits and therefore the implementation team had to overcome the following 3 key challenges: (1) developing technical integration protocols from the ground up, (2) developing a process for patient identity verification prior to group session admission, and (3) reducing friction points in the workflows for patients and providers.

**Figure 1:**
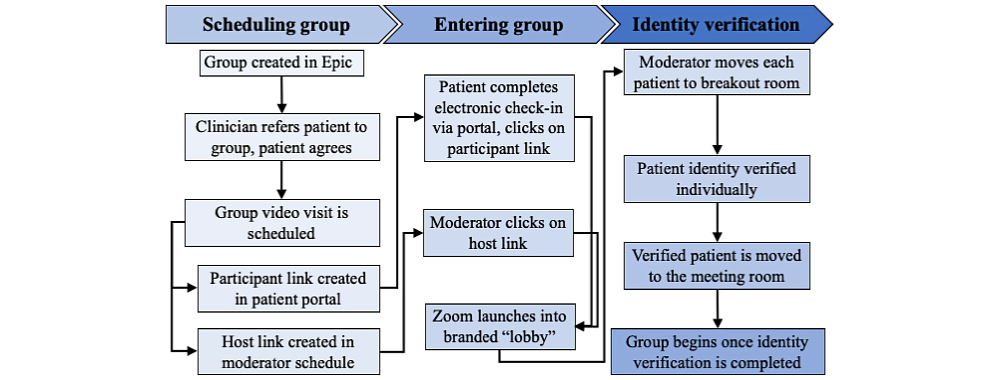
Administrative, clinical, and patient workflow for synchronous group video visits for health interventions, including scheduling, entering the group, and verifying patient identity.

Over approximately 6 weeks, beginning in April 2020, the WCH information technology (IT) team integrated Zoom with Epic in a manner that addressed all requirements, including seamless recording in the patient’s EMR (to facilitate documentation of patient care), automated creation and dissemination of meeting links (to reduce administrative and clinical burden), and secure patient identity verification. These factors would not have been possible if a nonintegrated system was used. A smooth administrative, clinical, and patient workflow was simultaneously developed (Figure 1). This echoed the scheduling workflow for individual video visits to reduce administrative burden and training requirements.

**Figure 2:**
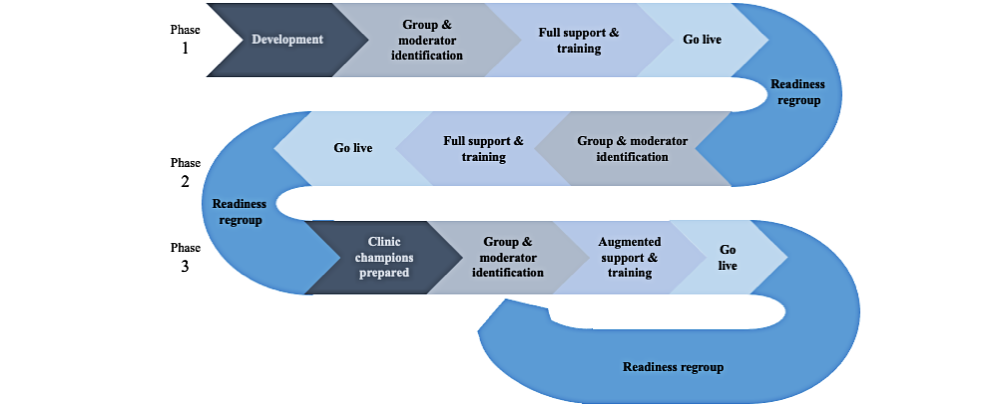
Overview of the 3-phased rollout of synchronous group video visits at Women’s College Hospital.

The first group video visit occurred on May 1, 2020, followed by a rapid 3-phased rollout to quickly scale group video visits across the hospital, allowing for iterative feedback and system improvements (Figure 2). Group moderators trained in the early stages of the rollout (with full support directly from the technical development team) acted as clinical champions to train their peers (using augmented training materials) by Phase 3. At the end of each phase, stakeholders conducted a debrief to determine the hospital’s readiness to move to the next phase of rollout and what, if any, technical or workflow changes needed to be made beforehand. Improvements over time included technical enhancements for improved audio and video quality and the development of training materials (Phase 1), clinical workflow enhancements for easier scheduling and patient identity verification (Phase 2), and minor format modifications to some groups to allow for logical and housekeeping considerations in the first group session (Phase 3). Across 5 hospital divisions, in the 6 months following the launch of synchronous video groups (ie, May to November 2020), 29 groups were run, involving 542 individually scheduled group sessions conducted with 767 unique participants across the hospital, representing diverse health concerns, ages, and care needs.

**The factors considered in selecting a sustainable technological platform to support the implementation of synchronous videoconferencing for group health interventions.**

1. Meets all legal and privacy regulations and best practices.
2. Supports group videoconferencing for ~25 participants.
3. Generates a single access link for moderators and patients.
4. Allows for the secure sharing of access links with moderators and patients.
5. Has admittance functionality.
6. Has a smooth log-in workflow for patients.
7. Allows for the documentation of completed appointments in the electronic medical record.

### Evaluation Approach

**Figure 3:**
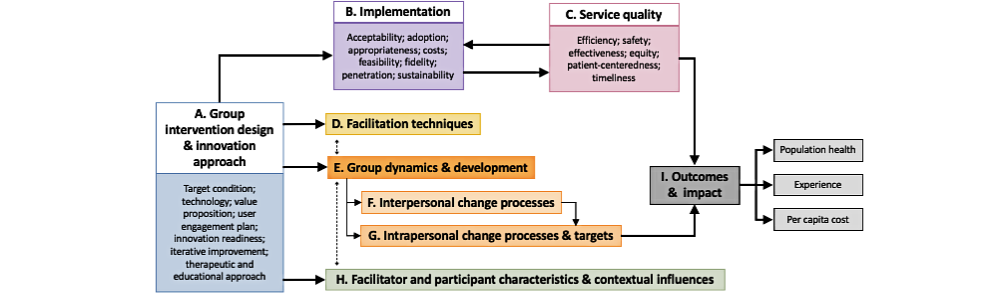
The Virtual Group Evaluation Framework to guide evaluations of synchronous group video visits, adapted from Proctor et al [13,14] and Borek et al [17,18], and using definitions from the Institute of Medicine [15] and the Quadruple Aim [16].

Much has been documented related to the evaluation of individual video visits, but there is a significant gap in the literature related to the evaluation of the implementation and quality of synchronous video visits to deliver group-based health interventions, as well as group functioning in a virtual context. It is our view that appropriate evaluation is of critical importance to developing an evidence-based understanding of the role that these innovations can and should play to enhance the delivery of health care. Given the novelty of institutionally driven, Epic-integrated implementation of synchronous video group interventions, we developed the Virtual Group Evaluation Framework (Figure 3) to guide its short- and long-term evaluation at our institution; this framework could be considered for use by other institutions who similarly transition to system-integrated sustainable models of delivering group health interventions virtually. This framework integrates an adapted model from Proctor et al [13,14] that enables the evaluation of programs at multiple stages of their innovation lifecycle by considering measures of implementation (acceptability, adoption, appropriateness, feasibility, fidelity, cost, penetration, and sustainability), quality of care [15] (safe, effective, patient-centered, timely, efficient, and equitable) and impact (defined by the Quadruple Aim [16] as an improved experience of providing and receiving care, better population health, and reduced per capita cost of health care), and the mechanisms of action in group-based interventions (MAGI) framework [17,18], which outlines the theoretical mechanisms that lead to change in group-based health interventions. The MAGI framework was developed based on an extensive mixed-methods multidisciplinary literature review and primary research.

The Virtual Group Evaluation Framework can be operationalized using a mixed-methods approach to data collection and analysis, which is valuable and commonly used to evaluate both virtual and group interventions, which are inherently complex [1]. The following 3 layers of data collection can provide a comprehensive evaluation of each component of the framework: (1) hospital-based data (eg, patient records and IT services) to measure the innovation approach, as well as components of implementation, service quality, and outcomes (Multimedia Appendix 1); (2) quantitative data from patients (eg, based on self-report quality improvement surveys) and facilitators to measure individual characteristics, group dynamics, and components of implementation, service quality, and outcomes (Multimedia Appendix 2); and (3) qualitative interviews with key stakeholders (eg, patients, facilitators, referring clinicians, and hospital administrators) to fill in the remaining gaps in knowledge and provide a more holistic and contextual understanding of the experience. Each layer of data can be collected and analyzed alone or in combination with other layers of data depending on the specific research question being addressed by the institution or individual departments conducting their own evaluation projects. Where possible, specific validated tools were identified for quantitative data collection to ensure that the data collected address all components of the framework and allow for consistency and comparability across the variety of group interventions that take place throughout the hospital (Multimedia Appendix 1 and Multimedia Appendix 2). Simultaneously, we believe that this evaluation approach is flexible enough to remain receptive to the needs of different groups and patient populations that they serve.

## Discussion

This paper describes the development and operationalization of group video visits to support the continued delivery of patient care in a virtual context, as well as a proposed strategy to guide the evaluation of their implementation, quality, and outcomes. To our knowledge, the experience of development and implementation of a clinically integrated and institutionally driven model of video-based group health interventions has not yet been described in the literature.

There are several key learnings that can be used to help inform similar undertakings by other institutions considering a similar approach. First, our focus in developing Epic-integrated synchronous video group visits was centered on the value proposition it could offer beyond the immediate context of COVID-19–related restrictions to care delivery. Therefore, we centered our efforts on addressing the needs of “tools, teams, and routines” to make meaningful improvements to care and quality of care [19]. This extended beyond the tool itself, to the design of administrative, clinical, and patient workflows, with rapid iterations at each stage of the phased rollout to make incremental improvements as feedback was received to ensure its long-term sustainable integration into the way group-based care is delivered at our institution.

Second, beyond ensuring patient care when in-person services are disrupted, it is vitally important to operationalize the virtual delivery of health care alongside approaches for evaluating its implementation, quality, and impact. Therefore, the development of an evaluation strategy that specifically considers the nuances of both virtual and group care will allow us to understand whether high-quality care that improves outcomes continues to be delivered to patients. The evaluation strategy developed as part of this work can be considered by other organizations pursuing similar innovations related to the delivery of virtual group health interventions and seeking to incorporate evaluation strategies. Our Virtual Group Evaluation Framework provides a theoretically driven yet practical way to support the short- and long-term evaluation of synchronous virtual group health interventions. It provides flexibility and adaptability to suit a variety of contexts (eg, group types, institutional capacities, and resources). Further, the evaluation strategy incorporates considerations of equity because although virtual health interventions may address some barriers to care, they simultaneously have the potential to exacerbate issues of inequity related to delivery and access. There are published examples of conceptually driven approaches for individual video visits [20], but none to our knowledge that consider the nuances of group health interventions when delivered in a virtual environment.

The limitations of this work should be noted. This paper describes the operationalization of group video visits in a tertiary care center with existing familiarity with video clinical visits and a high focus on innovation; therefore, this experience may not be broadly applicable to all centers, particularly those where the use of virtual care is more novel. However, we still feel that our experience in developing and operationalizing group video visits can be used to provide a guide for institutions looking to introduce such strategies. While the MAGI framework, which guides the evaluation of group process outcomes, has not yet been widely used, given its recent publication, nor has it been applied to group health interventions in a virtual setting, this work will add to the literature to address these gaps. Finally, the Virtual Group Evaluation Framework has not yet been used or validated in clinical virtual group interventions. Therefore, its utility and generalizability in this context need to be determined prior to more widespread use by other organizations. This will be explored as the framework is applied in practice; evaluation efforts at WCH that apply the use of this framework are underway.

Future work includes conducting evaluations using the Virtual Group Evaluation Framework to understand outcomes and gain a better understanding of group functioning in a virtual environment. Groups also need to be optimized to support the delivery of different types of group health interventions, such as movement-based groups (eg, exercise or yoga). Additionally, the experience of group health interventions through synchronous videoconferencing and results of evaluations should be used when considering how this type of care delivery fits into the postpandemic health system. If we can demonstrate its positive impact, it can be used to deliver care to those who might not otherwise be able to access the types of specialized care that our center provides due to geographic, social, or medical isolation. For example, some cardiac rehabilitation groups at WCH were able to operate over the summer months, a time when their target patient population may not be able to travel to the hospital to receive care due to high heat, which can exacerbate certain cardiac conditions and symptoms.

The rapid operationalization of virtual care has been a consistent feature in health care delivery during the COVID-19 pandemic, and one that is likely to have enduring effects on how care is delivered. The experience of our center in systematically operationalizing and delivering EMR-integrated synchronous video group health interventions is one such example that emerged based on need but was designed in a manner that has the potential to enhance how virtual care can be scaled and sustained. Its evaluation will provide the data needed to support its place in a postpandemic health system.

## Appendix

Multimedia Appendix 1 The operational approach to evaluating synchronous video group health interventions using hospital-based data.

Multimedia Appendix 2 The operational approach to evaluating synchronous video group health interventions using self-reported quantitative data.

## Abbreviations

EMR: electronic medical record
IT: information technology
MAGI: mechanisms of action in group-based interventions
WCH: Women’s College Hospital
WV: Women’s Virtual
