# Supplementary material for: Operationalizing and Evaluating Synchronous Virtual Group Health Interventions: Wide-Scale Implementation at a Tertiary Care Academic Hospital
Source: J Med Internet Res. 2022 Apr 7;24(4):e29841. doi: 10.2196/29841 (PMC9030913; doi:10.2196/29841)
Supplement: Multimedia Appendix 1 [file jmir_v24i4e29841_app1.docx]

## Multimedia Appendices

Multimedia Appendix, Table 1. The operational approach to evaluating synchronous video group health interventions using hospital-based data.

|  | **Definition** | **Suggested Data** |
| --- | --- | --- |
| **A. Group Intervention Design & Innovation Approach** |  |  |
| Group Intervention Design | Features of the design that may affect functioning of the group and its delivery | - Group purpose, duration and characteristics - Intended outcome and participants |
| **B. Implementation** |  |  |
| Acceptability | Perception that a service is satisfactory. | n/a  *See: Multimedia Appendix, Table 2* |
| Adoption | The intention or action to use an evidence-based practice. | - Patient-level indicators of active use - Number of groups (per hospital division, overall) |
| Appropriateness | Perceived fit of a practice for a given setting or to address a problem. | - 1:1 clinical visits and purpose |
| Costs | The cost impact of an implementation effort. | - Use of technical support - Procurement, implementation, maintenance costs |
| Feasibility | The extent to which an innovation can be successfully used. | - Use of technical support |
| Fidelity | The extent to which implementation occurs as it was intended to. | n/a  *See: Multimedia Appendix, Table 2; qualitative data can also be used* |
| Penetration | The integration of a practice within a setting *(Note: early penetration only)* | - Number of groups (per hospital division, overall) |
| Sustainability | The extent to which a new innovation is maintained as a part of ongoing operations. | - Indicators of penetration over time |
| **C. Service Quality** |  |  |
| Efficiency | Avoidance of waste. | n/a  *See: Multimedia Appendix, Table 2; qualitative data can also be used* |
| Safety | Avoidance of harm from care that is intended to help. | - Adverse events; deterioration in clinical status |
| Effectiveness | Provision of evidence-based services to those who can benefit. | - Change in relevant clinical status |
| Equity | Providing care that does not vary in quality due to socioeconomic, demographic, or personal characteristics. | - Patient age, sex, gender, postal code, language |
| Patient-Centred | Provision of care that is respectful and responsive to patient needs. | n/a  *Qualitative data only* |
| Timeliness | Low wait times or delays. | - Interval between group referral and first session |
| **Group-Based Measures** |  |  |
| D. Facilitation Techniques | Methods used by facilitator(s) to deliver sessions, facilitate group dynamics and initiate planned change process. | n/a  *See: Multimedia Appendix, Table 2; qualitative data can also be used* |
| E. Group Dynamics and Development | Processes used to describe group functioning and how it changes over time. |  |
| F. Interpersonal Change Processes | Change processes that operate in, and are unique to a social context such as a group intervention. |  |
| G. Intrapersonal Change Processes | Change processes and psychological targets that operate within an individual. |  |
| H. Facilitator and Participant Characteristics, Context | Factors external to the group that may influence (or be influenced by) what occurs in the group. | - Age, sex, gender, postal code |
| **I. Impact** |  |  |
| Clinical outcomes | The targeted change in physical or psychological well-being | - Change in health status |
| Overall experience | Overall perception of intervention | n/a  *See: Multimedia Appendix, Table 2; qualitative data can also be used* |
