# Supplementary material for: Operationalizing and Evaluating Synchronous Virtual Group Health Interventions: Wide-Scale Implementation at a Tertiary Care Academic Hospital
Source: J Med Internet Res. 2022 Apr 7;24(4):e29841. doi: 10.2196/29841 (PMC9030913; doi:10.2196/29841)
Supplement: Multimedia Appendix 2 [file jmir_v24i4e29841_app2.docx]

## Multimedia Appendices

Multimedia Appendix, Table 2. The operational approach to evaluating synchronous video group health interventions using self-report quantitative data.

|  | **Definition** | **Suggested Measurements^a^** | |
| --- | --- | --- | --- |
| **A. Group Intervention Design & Innovation Approach** |  |  | |
| Group Intervention Design | Features of the design that may affect functioning of the group and its delivery | n/a  *See: Multimedia Appendix, Table 1* | |
| **B. Implementation** |  |  | |
| Acceptability | Perception that a service is satisfactory. | Patient | - Acceptability of group: Satisfaction with Therapy and Therapist Scale – Revised (STTS-R)[1] - Acceptability of technology: Questions on satisfaction with virtual platform |
|  |  | Provider | - Acceptability of technology: Questions on satisfaction with virtual platform (e.g. With regards to the technology, rate your experience from 1-10) |
| Adoption | The intention or action to use an evidence-based practice. | n/a  *See: Multimedia Appendix, Table 1* | |
| Appropriateness | Perceived fit of a practice for a given setting or to address a problem. | Patient | - STTS-R[1] |
| Costs | The cost impact of an implementation effort. | Patient & Provider | - Costs and benefits of participation (e.g. Did the virtual group save you time?) or facilitation (e.g. How much preparation time did you take for virtual group vs in-person?) |
| Feasibility | The extent to which an innovation can be successfully used. | Patient & Provider | - Use of training materials - Comfort with technology (e.g. How would you rate your comfort with using technology in your day-to-day life?) |
| Fidelity | The extent to which implementation occurs as it was intended to. | Provider | - Treatment adherence logs |
| Penetration | The integration of a practice within a setting *(Note: early penetration only)* | n/a  *See: Multimedia Appendix, Table 1* | |
| Sustainability | The extent to which a new innovation is maintained as a part of ongoing operations. |  |  |
| **C.** **Service Quality** |  |  | |
| Efficiency | Avoidance of waste. | Patient & Provider | - Costs and benefits of participation or facilitation |
| Safety | Avoidance of harm from care that is intended to help. | Patient | - Self-reported clinical outcomes - Confidence in and concerns about virtual groups (e.g. Did you have concerns about your privacy, confidentiality or safety?) |
|  |  | Provider | - Confidence in and concerns about virtual groups |
| Effectiveness | Provision of evidence-based services to those who can benefit. | Patient | - Self-report clinical outcomes - STTS-R (Q13)[1] |
| Equity | Providing care that does not vary in quality due to socioeconomic, demographic, or personal characteristics. | Patient | - Income - Education - Access to technology |
| Patient-Centred | Provision of care that is respectful and responsive to patient needs. | n/a  *Qualitative data only* | |
| Timeliness | Low wait times or delays. | n/a  *See: Multimedia Appendix, Table 1* | |
| **Group-Based Measures** |  |  | |
| **D. Facilitation Techniques** | Methods used by facilitator(s) to deliver sessions, facilitate group dynamics and initiate planned change process. | Patient | - STTS-R (Subscale 2)[1] |
|  |  | Provider | - Group observation - Example: Trainer Behaviour Scale[2,3] |
| **E. Group Dynamics and Development** | Processes used to describe group functioning and how it changes over time. | Patient | - May vary based on type of group. - Examples: The Group Questionnaire[4] *(for therapeutic groups)*, Community Connection Scale[5] *(for educational groups)* |
| **F. Interpersonal Change Processes** | Change processes that operate in, and are unique to a social context such as a group intervention. | Patient | - Should vary based on targeted change processes of group. - Example: Social Provisions Scale, Short Version (SPS-10)[6] |
| **G. Intrapersonal Change Processes** | Change processes and psychological targets that operate within an individual. | Patient | - Should vary based on targeted change processes of group. - Example: New General Self-Efficacy (NGSE) Scale[7] |
| **H. Facilitator and Participant Characteristics, Context** | Factors external to the group that may influence (or be influenced by) what occurs in the group. | Patient & Provider | - Socio-demographics - Comfort with technology. Example: eHealth Literacy Scale (eHEALS)[8] |
| **I. Impact** |  |  |  |
| Clinical outcomes | The targeted change in physical or psychological well-being | Patient | - Self-reported clinical outcomes |
| Overall experience | Overall perception of intervention | Patient | - STTS-R[1] - Questions on overall experience (e.g. Please rate your overall experience from 1-10) |
|  |  | Provider | - Questions on overall experience and satisfaction |
| Legend: | ^a^Measurement tools are suggested examples and should be used after considering its appropriateness for the group under study. | | |

## Multimedia Appendices References

1. Oei TPS, Green AL. The satisfaction with therapy and therapist scale-revised (STTS-R) for group psychotherapy: psychometric properties and confirmatory factor analysis. *Prof Psychol Res Pract*; 2008;39(4), 435-442. doi:10.1037/0735-7028.39.4.435

2. Borek AJ, Smith JR, Greaves CJ, et al. Developing and applying a framework to understand mechanisms of action in group-based, behaviour change interventions: the MAGI mixed-methods study. *Effic Mech Eval*; 2019;6(3):1-162. doi:10.3310/eme06030

3. Bolman L. Some effects of trainers on their t groups. *J Appl Behav Sci*; 1971;7(3):309-325. doi: 10.1177/002188637100700303

4. Thayer SD, Burlingame GM. The validity of the group questionnaire: Construct clarity or construct drift? *Gr Dyn*; 2014;18(4), 318-332. doi:10.1037/gdn0000015

5. Rovai AP. Development of an instrument to measure classroom community. *Internet High Educ*; 2002;5(3):197-211. doi: 10.1016/S1096-7516(02)00102-1

6. Orpana HM, Lang JJ, Yurkowski K. Validation of a brief version of the social provisions scale using Canadian national survey data. *Heal Promot Chronic Dis Prev Canada*; 2019;39(12):323-332. doi:10.24095/hpcdp.39.12.02

7. Chen G, Gully SM, Eden D. Validation of a new general self-efficacy scale. *Organ Res Methods*; 2001;4(1):62-83. doi:10.1177/109442810141004

8. Norman CD, Skinner HA. eHEALS: The eHealth literacy scale. *J Med Internet Res*; 2006;8(4):e27. doi:10.2196/jmir.8.4.e27
